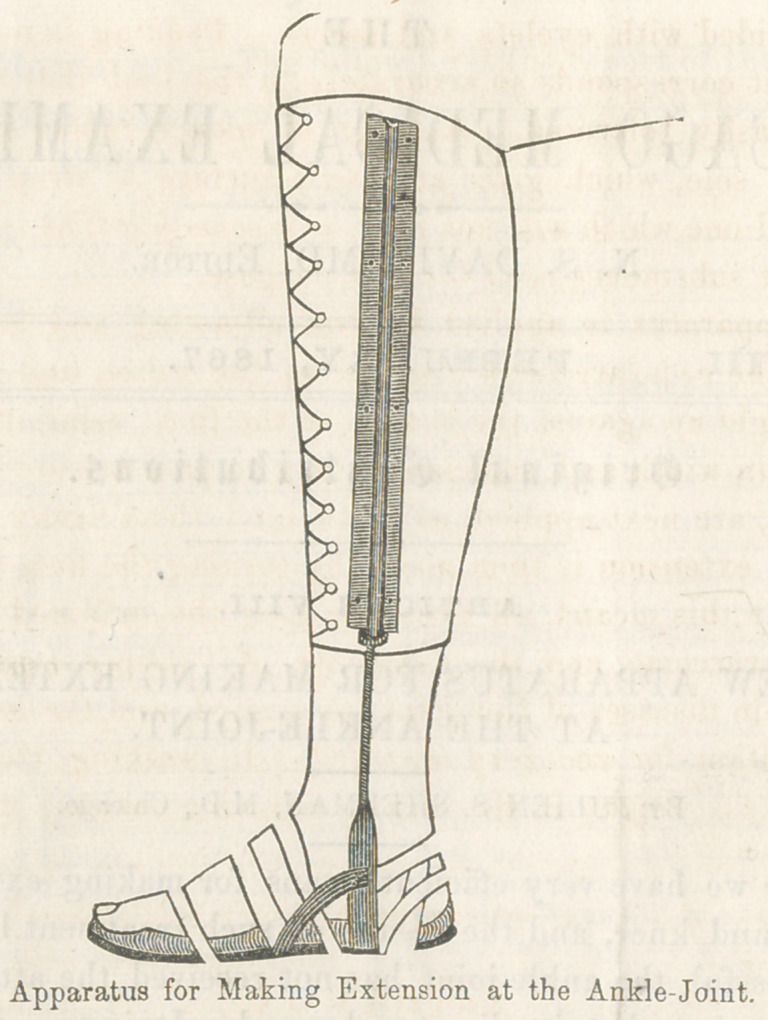# A New Apparatus for Making Extension at the Ankle-Joint

**Published:** 1867-02

**Authors:** Julien S. Sherman

**Affiliations:** Chicago


### CHICAGO MEDICAL EXAMINER.

N. S. DAVIS, MD, Editor.

VOL. VIII. FEBRUARY, 1867. NO. 2.

### Original Contributions.

ARTICLE VIII.

### A NEW APPARATUS FOR MAKING EXTENSION AT THE ANKLE-JOINT.

By JULIEN S. SHERMAN, M.D., Chicago.

While we have very efficient means for making extension at the hip and knee, and the results of such treatment have been so successful, the ankle-joint has not received the attention, in this direction, that its diseases demand. It is as necessary to remove pressure from an inflamed ankle as from an inflamed hip or knee-joint. The application is, however, more difficult, and, for that reason, has not been used as frequently as desirable. Dr. Sayre, some time since, devised an instrument for this purpose, in which both the extension and counter-extension were made by adhesive straps. These are liable to become torn and detached, requiring frequent renewal, especially those upon which the counter-extending force is brought to bear. At the same time, the patient is required, in most instances, to use crutches.

The annexed cut represents an apparatus which I have lately been using, and have found most satisfactory in every respect. The counter-extension is made by means of a *leather socket*, upon the same principle that I represented for the knee-joint, in the January No. of the Examiner.

Apparatus for Making Extension at the Ankle-Joint.

In order to construct it properly, a cast of the patient's leg, from the knee to the ankle, should be taken in plaster of Paris. It is not necessary to include the foot. A piece of sole leather should then be soaked in water, until perfectly soft and pliable, and then moulded and firmly tied upon the cast. It should remain in this condition until thoroughly dry, when it will be found to form a strong socket, *exactly* corresponding to the shape of the leg from which the cast was taken. A steel sole is then cut, the shape of the bottom of the foot, being somewhat narrower beneath the instep and heel. From this, and riveted to its under surface, rises, on either side, a steel rod, bent to the shape of the limb; upon its upper two-thirds a screw is cut. This rod is received into a tube, which is attached to the socket. The screw carries a nut, by which the extension is regulated. The rod is braced to the sole by an additional strip of steel on either side. The socket is lined with buckskin and provided with eyelets and lacers. Padding is not necessary, as it corresponds so accurately to the limb that the pressure is easily tolerated. A layer of *sponge rubber* is placed upon the sole, which gives an elastic surface to strap the foot upon, and one which will not *pack or become hard*, as is the case with most substances used for this purpose.

The apparatus is applied by first adjusting and lacing the socket; the rods are next inserted in the tubes, and the steel sole brought up against the bottom of the foot; adhesive straps, an inch in width and sufficiently long to pass around the foot and heel, are next applied, so as to bind them firmly down to the sole; extension is then made, by turning the nuts upon the rods. By this means, all pressure upon the inflamed surfaces will be overcome, and the deformity of "talipes equinus," so frequent in diseases of this joint, prevented, and the best possible condition for recovery obtained. In walking, the patient bears all his weight in the socket, relieving entirely the ankle-joint.

## Figures and Tables

**Figure f1:**